# Chronic Intermittent Hypoxia‐Induced Neural Injury: Pathophysiology, Neurodegenerative Implications, and Therapeutic Insights

**DOI:** 10.1111/cns.70384

**Published:** 2025-04-22

**Authors:** Nan‐Nan Jia, Meng‐Fan Yao, Chun‐Xue Zhu, Mei‐Juan He, Hai‐Feng Zhu, Zun‐Yu Chen, Han‐Peng Huang, Chen Qiao

**Affiliations:** ^1^ The Affiliated Hospital of Jiangsu University Jiangsu University Zhenjiang Jiangsu China; ^2^ Department of Respiratory and Critical Care Medicine The Affiliated Hospital of Jiangsu University Zhenjiang Jiangsu China; ^3^ College of Pharmacy Jiangsu University Zhenjiang Jiangsu China

**Keywords:** chronic intermittent hypoxia, glial cells, neural injury, neuroinflammation, obstructive sleep apnea‐hypopnea syndrome

## Abstract

Obstructive sleep apnea‐hypopnea syndrome (OSAHS) is a sleep‐related respiratory disorder that poses a global threat to human health. Chronic intermittent hypoxia (CIH) is its main pathological feature. With the advancements in medical research, the study of CIH‐induced neural injury has gained increasing attention. Studies have shown that CIH can lead to or aggravate neuroinflammation and apoptosis by increasing blood–brain barrier (BBB) permeability, promoting oxidative stress, activating glial cells, and triggering multiple signaling pathways, ultimately resulting in neural injury. These processes contribute to the development of Alzheimer's disease, Parkinson's disease, and stroke. This review aims to summarize the progress in CIH‐induced neural injury and explore various underlying mechanisms, with the goal of providing new insights for the development of therapeutic interventions targeting CIH‐related neural damage.

## Graphical abstract

Given the established association between obstructive sleep apnea‐hypopnea syndrome (OSAHS) and neurodegenerative pathogenesis, this review systematically investigates the mechanisms by which chronic intermittent hypoxia (CIH) contributes to OSAHS‐related neural injury. By exploring key pathways such as oxidative stress, inflammatory reaction, and blood–brain barrier disruption, we aim to uncover potential therapeutic targets. These insights may inform the development of novel interventions to mitigate CIH‐induced neurological damage, offering new avenues for treatment.

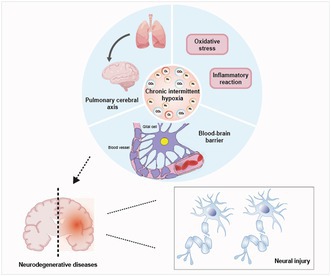

## 1 Introduction

Obstructive Sleep Apnea‐Hypopnea Syndrome (OSAHS) is a type of sleep‐related breathing disorder characterized by the complete cessation of oronasal airflow for ≥ 10 s (apnea) or a reduction in airflow by ≥ 30% accompanied by a decrease in oxygen saturation of ≥ 3% or arousal (hypopnea) during sleep [1]. This contributes to various health issues. OSAHS is highly prevalent worldwide, and the latest research shows that its incidence is rising, affecting 5%–15% of the global population [2]. The prevalence increases with age, making OSAHS a significant global public concern [2, 3]. The risk factors of OSAHS include body weight, gender, age, upper airway soft tissue, and enlarged craniofacial abnormalities. Additionally, lifestyle factors and clinical symptoms like habitual snoring, nocturnal awakenings, nocturnal polyuria, gastroesophageal reflux, and morning headache are associated with OSAHS [1]. The hallmark pathological feature of OSAHS is the repeated partial or complete obstruction of the upper airway during sleep, leading to intermittent hypoxia and systemic inflammation, which increases the risk of cardiovascular and cerebrovascular diseases [4].

Clinical diagnosis involves a comprehensive history, clinical examination, home sleep apnea test (HSAT), and polysomnography (PSG) [5, 6]. The diagnostic criteria are apnea‐hypopnea index (AHI) ≥ 5 times/h [7]. The primary goal of OSAHS treatment was to reduce apnea‐hypopnea events, reduce the risk of complications, and minimize healthcare costs. Current treatments include lifestyle changes, continuous positive airway pressure (CPAP), mandibular advancement devices, surgery, and pharmacotherapy [7]. Although CPAP is the gold standard for the treatment, its high cost and poor patient compliance often decrease its efficacy and increase side effects, worsening disease complications and lowering patients' quality of life [8]. Pharmacological treatments are gaining attention, with studies showing that medicines like atomoxetine combined with oxybutynin, donepezil, sodium oxybate, trazodone, and modafinil have demonstrated positive effects on alleviating intermittent hypoxia in OSAHS [9, 10, 11].

Chronic intermittent hypoxia (CIH) is a hallmark of OSAHS. It involves repeated episodes of apnea and hypoventilation due to upper airway collapse during sleep, resulting in periodic decline and recovery of blood oxygen levels. This periodic process of hypoxia and reoxygenation affects various body systems, especially the nervous system [2]. Studies have shown a close association between OSAHS and neurological damage, particularly in cognitive impairment and hippocampal injury [12, 13]. CIH exacerbates neural injury by inducing neuroinflammation and apoptosis, increasing blood–brain barrier permeability, disrupting the noradrenergic system, and impairing the function of neuroprotective factors [14, 15]. These effects indicate the extensive negative regulatory influence of CIH on the nervous system, highlighting the need for effective treatment and intervention measures to mitigate its damage. Elucidating the pathophysiological relationship between CIH and neural injury holds significant clinical implications. This understanding may facilitate early diagnosis and timely intervention in patients with OSAHS, while also providing an evidence‐based foundation for developing targeted public health strategies and optimizing healthcare resource allocation.

Neural injury may lead to a variety of dysfunctions, such as sensory loss, movement disorders, cognitive problems, and emotional changes [16, 17, 18]. Research indicates that OSAHS increases the risk of neurodegenerative diseases, including Alzheimer's disease (AD) and Parkinson's disease (PD) [19, 20]. CIH, as induced by OSAHS, has been found to lower neurological function scores and neuron survival rates, while also upregulating proteins associated with apoptosis and neuroinflammation. The proteins include caspase‐3 (CASP3), Bcl‐2‐associated X protein (Bax), interleukin‐1β (IL‐1β), interleukin‐6 (IL‐6), tumor necrosis factor‐α (TNF‐α), and nuclear transcription factor‐κB (NF‐κB) [14, 21]. Notably, the effects of OSAHS differ between adults and children. In adults, OSAHS leads to brain structural changes, cognitive decline, autonomic dysfunction, and emotional and behavioral abnormalities. In children, it primarily results in developmental delay, abnormal behavior, attention issues, and learning disabilities [22]. While the precise mechanism has not been fully clarified, previous studies suggest that OSAHS may increase the blood–brain barrier permeability, leading to cerebral edema and brain injury. Additionally, it may impair cognitive function by affecting the hippocampal neurons and exacerbate neuroinflammation and apoptosis by activation of the recombinant activating transcription factor 4 (ATF4)/C/EBP‐homologous protein (CHOP) signaling pathway, or by intensifying neural injury through the hypoxia‐inducible factor‐1α (HIF‐1α) and apoptosis‐associated speck‐like protein containing a CARD (ASC) signaling pathways [21, 23].

CIH significantly increases the risk of neurodegenerative diseases in patients with OSAHS. OSAHS is now widely recognized as a risk factor for cognitive decline, AD, PD, cerebrovascular diseases, affective disorders, and various other neurological diseases [15, 24, 25]. However, the specific mechanism underlying the complex relationship between CIH caused by OSAHS and these neural injuries remains unclear. This paper aims to provide a comprehensive review of the existing research on the association between OSAHS and neural damage, while also suggesting potential directions for future research into the mechanisms and therapeutic strategies for OSAHS‐related neural injury.

## 2 Mechanisms of CIH‐Induced Neuronal Injury

### 2.1 The Role of CIH in Neuronal Damage Capability

#### 2.1.1 Impact of CIH on Neuronal Injury

**FIGURE 1:**
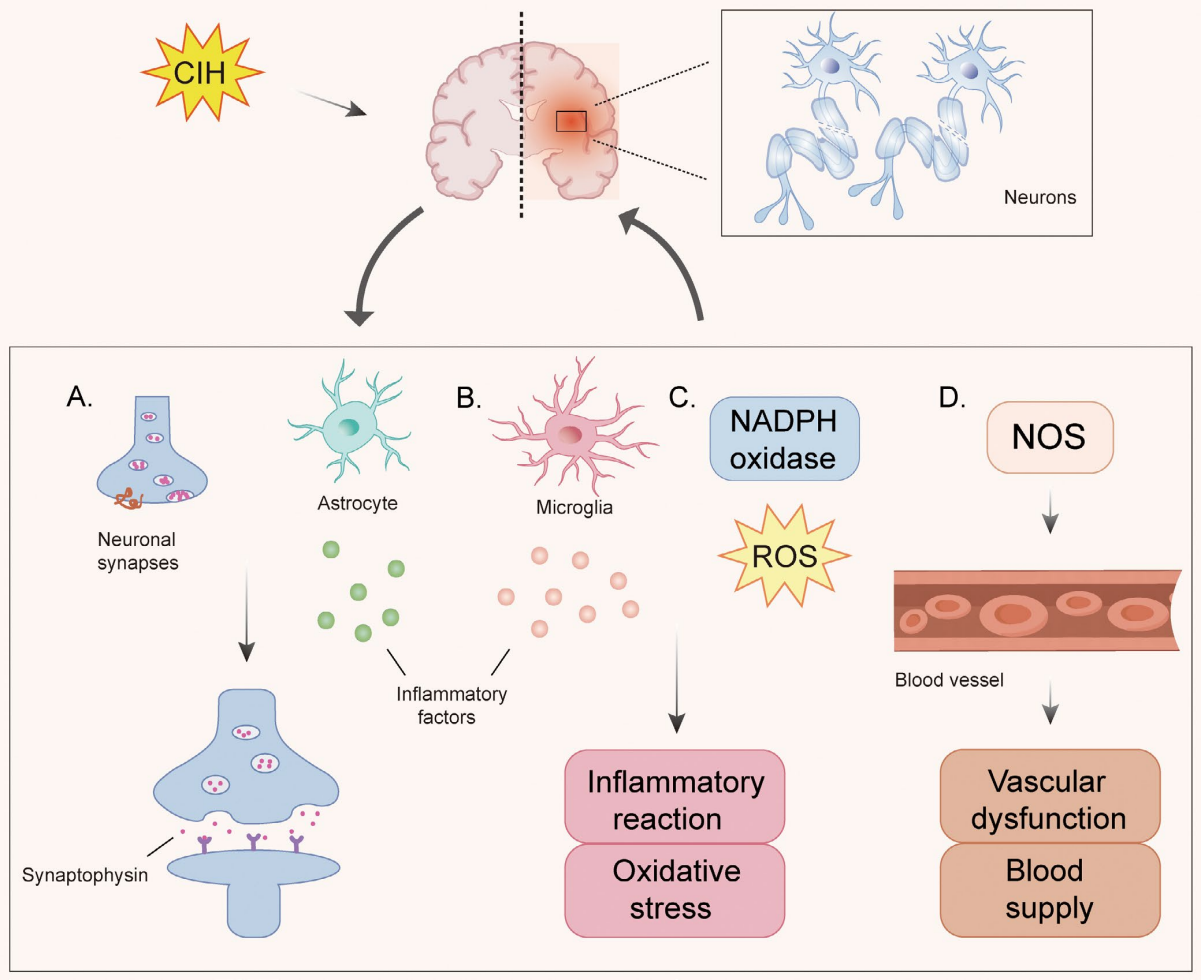
The underlying mechanism of CIH‐induced neuronal damage. (A) CIH can exert an impact on the structure and function of neuron synapses and thereby cause damage to neurons by inhibiting the synthesis of synaptophysin; CIH can activate astrocytes to release inflammatory factors that affect the formation and stability of neuronal synapses, thereby damaging neurons. (B) CIH can activate microglia to release inflammatory factors, trigger inflammatory reactions and oxidative stress, and then damage neurons. (C) CIH can enhance NADPH enzyme activity, promote the production of ROS, trigger inflammatory reactions and oxidative stress, and then damage neurons. (D) CIH induces NOS activation via multiple signaling pathways, resulting in vascular endothelial dysfunction, compromised blood flow, and subsequent neuronal injury.

The impact of CIH on neuronal damage is multifaceted and can be summarized as follows: First, CIH contributes to neuronal damage by altering the formation and function of neuronal synapses. It not only modulates the synaptic structure and function by regulating synaptophysin expression but also affects the formation and stability via astrocyte activation and the release of inflammatory factors, ultimately leading to neuronal damage (Figure 1A) [25, 26]. Second, CIH triggers neuronal damage via inflammatory reactions. It can directly affect microglia or indirectly influence neurons via peripheral or other CNS cells, provoking inflammatory responses that harm neurons (Figure 1B) [27, 28]. Third, CIH induces neuronal damage by promoting oxidative stress. It activates NADPH oxidase and increases the generation of reactive oxygen species (ROS), leading to aggravated oxidative damage and subsequently affecting nerve cells (Figure 1C) [28]. Lastly, CIH can cause neuronal damage by disrupting the blood supply. It reduces the synthesis and release of nitric oxide (NO) by impairing the expression and activity of endothelial nitric oxide synthase (NOS), leading to vascular dysfunction, compromised blood flow, and subsequently causing neuronal damage (Figure 1D) [29]. In summary, the mechanisms underlying CIH‐induced neuronal damage are complex. Therefore, timely treatment and intervention are particularly important for patients with neuronal damage caused by CIH.

#### 2.1.2 The Mechanism of CIH in Neuronal Survival and Its Link to Neurological Disease Development

The mechanisms by which CIH affects neurons are multifaceted, including alterations in neuronal structure and function, inflammatory reactions, oxidative stress, and abnormal blood supply.

First, CIH impacts neurons by modifying their structure and function. Synaptophysin, a fibrous phosphoprotein located in axon terminals, plays a crucial role in information transmission between neurons through phosphorylation. It is also an important marker for synapse formation and synaptogenesis [30]. Under CIH conditions, the calcium‐sensing receptor (CaSR) is activated, triggering the CaSR‐Protein kinase C (PKC)‐Extracellular regulated protein kinases 1/2 (ERK1/2) pathway. This cascade leads to a regulation in synaptophysin expression, which impairs synaptic function and subsequently causes neuronal damage [26]. Consequently, quantitative assessment of synaptophysin expression levels serves as a valuable tool for both evaluating the extent of neuronal injury and potentially identifying reliable biomarkers for the early detection of CIH‐induced neural damage. This dual utility underscores the clinical significance of synaptophysin monitoring in CIH‐related neurological disorders.

Second, CIH affects neurons through inflammatory responses and oxidative stress. The P_2_X_7_ receptor, a nonspecific cation channel, activates the nucleoside binding oligomerization domain‐like receptor family (NLRP3) inflammasome, which processes and activates the caspase‐1 channel, leading to the maturation of pro‐IL‐1β and pro‐IL‐18 and triggering neuroinflammation [31]. CIH can activate the P_2_X_7_ receptor, increasing the expression of inflammatory factors, NF‐κB and NADPH oxidase 2, resulting in oxidative stress and neuronal damage [32]. Furthermore, ferroptosis, an iron‐dependent form of oxidative cell death, occurs when iron ions react with oxidants such as hydrogen peroxide (H_2_O_2_) to produce stronger oxidants. Under hypoxic conditions, iron metabolism imbalances will lead to ferroptosis, intensify oxidative stress, and damage neurons [33]. Additionally, damage‐associated molecular patterns (DAMPs) are endogenous molecules released from damaged cells or tissues in response to injury, hypoxia, or stress. DAMPs are recognized by pattern recognition receptors (PRRs) of the immune system, triggering immune responses and inflammatory responses [34]. In CIH conditions, increased blood–brain barrier permeability allows DAMPs to enter the central nervous system (CNS), activating microglia and releasing inflammatory factors such as TNF‐α and IL‐1β, thereby provoking neuroinflammation [35].

Third, CIH can also cause neuronal damage through Toll‐like receptors (TLRs), P38 mitogen‐activated protein kinase (MAPK)/NF‐κB, or c‐Jun N‐terminal kinase (JNK)/NF‐κB signaling pathways [21, 23, 28].

Lastly, CIH adversely impairs neurons by disrupting the blood supply. Nitric oxide synthase (NOS) is an essential enzyme found in endothelial cells, macrophages, and neurons, catalyzing the conversion of L‐arginine into nitric oxide (NO). NO, a gaseous signaling molecule, freely diffuses across cell membranes to facilitate intercellular communication. NO plays a pivotal role in regulating vascular tone within vascular endothelial cells through a well‐characterized signaling cascade. Specifically, NO activates soluble guanylyl cyclase (sGC), increasing intracellular cyclic guanosine monophosphate (cGMP) levels. This elevation in cGMP triggers the relaxation of vascular smooth muscle cells, resulting in vasodilation and subsequent enhancement of blood flow to target tissues [36]. There are three main subtypes of NOS: neuronal nitric oxide synthase (nNOS), endothelial nitric oxide synthase (eNOS), and inducible nitric oxide synthase (iNOS). nNOS is predominantly expressed in neurons and facilitates cellular communication, while eNOS is found mainly in endothelial cells. eNOS, a fundamental constituent of the neurovascular unit, serves as a crucial regulator of cerebrovascular homeostasis. Through its catalytic production of NO, eNOS orchestrates vasodilation and modulates cerebral blood flow, thereby maintaining optimal perfusion pressure. This neurovascular coupling mechanism is indispensable for sustaining neuronal viability, synaptic plasticity, and the delicate homeostasis of the cerebral microenvironment [37]. Conversely, iNOS is expressed in response to injury or pathological stimuli such as ischemia or hypoxia, generating large amounts of NO and inducing oxidative stress [38]. CIH activates iNOS while reducing eNOS production, leading to abnormal vasoconstriction and impaired blood supply to neurons [29].

CIH is also closely associated with the progression of various neurodegenerative diseases. Previous studies have shown that CIH may contribute to the pathological process of AD by influencing β‐amyloid metabolism, promoting Tau protein phosphorylation, reducing brain‐derived neurotrophic factor (BDNF) expression, and inducing neuronal apoptosis [20, 39, 40]. In PD, CIH exacerbates neuronal damage through α‐synuclein accumulation [41]. Moreover, in multiple sclerosis (MS), CIH may impair the regenerative capacity of myelin sheaths [42]. Beyond the mechanisms mentioned, CIH is implicated in the development of AD, PD, MS, Huntington's disease (HD), and Amyotrophic Lateral Sclerosis (ALS) by enhancing oxidative stress and neuroinflammatory responses [39, 40, 43]. However, further research is required to elucidate how CIH specifically affects types of neurons and to elucidate the precise mechanisms that link alterations to distinct neurological disorders.

#### 2.1.3 Advances in Research on CIH Regulation of Neurons

Early studies have shown that oxidative stress and inflammatory response are common features of neurodegenerative diseases and are associated with OSAHS [44]. This phenomenon has attracted widespread attention from researchers, who have begun to explore its potential causes and mechanisms. Over years of research, CIH, the main pathological feature of OSAHS, has been identified as closely associated with neuronal damage. CIH impacts neuronal function by altering the structure and function of neuronal synapses, enhancing oxidative stress and inflammation, and impairing blood supply to neurons, all of which contribute to neuronal injury. CIH‐mediated neuronal injury exhibits regional specificity, predominantly affecting brain areas integral to cognitive processing and emotional regulation, particularly the hippocampus, prefrontal cortex, and amygdala [45]. CIH‐induced neuronal damage is reversible to some extent. For instance, continuous positive airway pressure (CPAP) treatment can partially restore the structure and function of the hippocampus [46]. As understanding of the mechanism behind CIH‐induced neuronal damage has deepened, researchers have turned to developing strategies to alleviate or reverse this damage. These studies enhance our understanding of CIH's impact on neurons and offer new insights into the prevention and treatment of related neurological diseases.

### 2.2 The Effects and Regulatory Mechanisms of CIH on Glial Cells Within CNS

#### 2.2.1 Astrocytes

**FIGURE 2:**
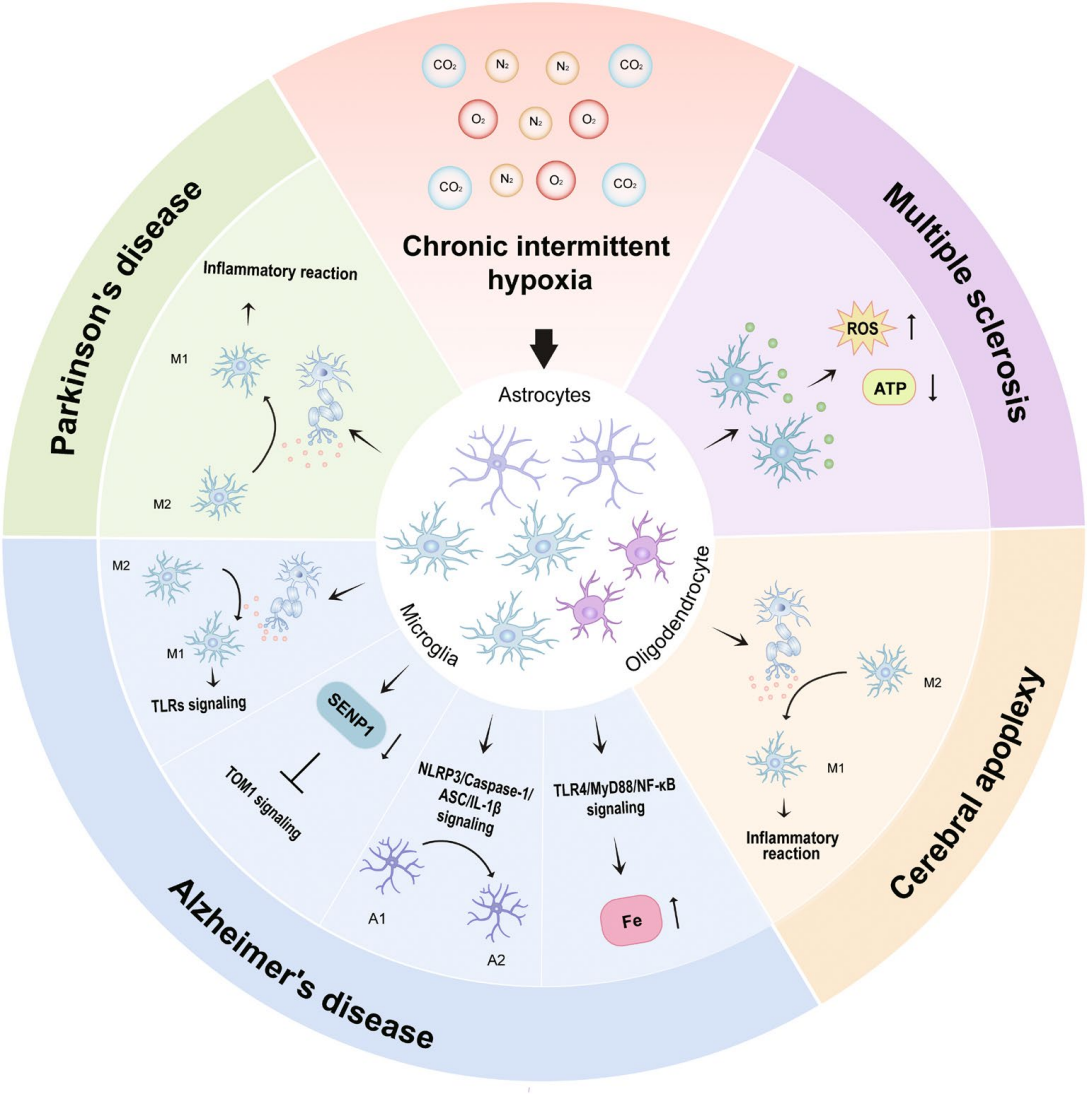
CIH modulates glial cells in the CNS through various mechanisms. CIH may be involved in the pathogenesis of Parkinson's disease and cerebral apoplexy by inducing the release of DAMPs from neuronal cells to regulate the M1/M2 phenotypic switch in microglia and trigger neuroinflammation. CIH can regulate the M1/M2 phenotypic transition of microglia by inducing the release of DAMPs, activate the TLRs signaling pathway, inhibit the TOM1 signaling pathway by reducing the level of SENP1, regulate the A1/A2 phenotypic transition of astrocytes by activating the NLRP3/Caspase‐1/ASC/IL‐1β pathway, and promote iron accumulation in microglia by activating the TLR4/MyD88/NF‐κB pathway, thus participating in the pathogenesis of AD. CIH may increase the production of reactive oxygen species in oligodendrocytes and reduce the production of ATP by activating microglia‐induced oxidative stress, thus contributing to the pathogenesis of multiple sclerosis.

Astrocytes are the most abundant cell type in the mammalian brain, exhibiting complex morphology. Mature astrocytes can interact with thousands of synapses and form connections with other astrocytes, occupying distinct spatial domains in the brain. They play vital roles in the development and functioning of the nervous system, including nourishing and supporting neurons, facilitating rapid nerve impulse conduction by enwrapping axons, and regulating synaptic connectivity and efficacy [47]. CIH has significantly affected astrocytes in the CNS through various regulatory mechanisms. For example, in the nucleus of the solitary tract (NTS) region, CIH can induce inflammation and neural overactivity, which subsequently activates astrocytes in the NTS, contributing to the pathogenesis of hypertension [48]. However, the precise mechanism is still unclear, warranting further investigation. Under pathological conditions, resting astrocytes will transform into reactive astrocytes, classified into A1 (neurotoxic) and A2 (neuroprotective) types. CIH has been shown to modulate the A1/A2 phenotype shift of astrocytes, contributing to the pathogenesis of AD by activating the NLRP3/Caspase‐1/ASC/IL‐1β signaling pathway (Figure 2) [49]. Understanding the molecular mechanism by which CIH affects astrocytes could lead to the development of novel interventions to mitigate CIH‐induced damage.

#### 2.2.2 Microglia

Microglia are resident macrophage‐like innate immune cells in the CNS with memory‐like properties. They interact with various other CNS types, including neurons, astrocytes, and oligodendrocytes. Microglia perform important physiological functions, such as monitoring the CNS microenvironment, phagocytosis, and neuronal support [35]. In the era of multiomics, it has been discovered that genetic risk factors for neurodegenerative and neuropsychiatric diseases such as AD, PD, schizophrenia, autism, and MS are primarily expressed by microglia [50]. The effects of CIH on microglia and its regulatory mechanisms are multifaceted. For example, CIH induces CNS cell damage, releasing DAMPs, which activate microglia through PRRs, leading to mitochondrial dysfunction, inflammation, endoplasmic reticulum stress, and neuronal dysfunction [28]. CIH can also drive the transformation of microglia from the anti‐inflammatory M2 phenotype to the pro‐inflammatory M1 phenotype, activating the TLRs signaling pathway and increasing the release of inflammatory factors, which in turn causes neuronal damage [28, 51]. Additionally, CIH promotes the expression of hepcidin via the TLR4/MyD88/NF‐κB signaling pathway. This results in iron accumulation in microglia, exacerbating oxidative stress and neuronal damage [25]. Another factor, SENP1, a small ubiquitin‐like modifier (SUMO) specific protease 1, plays a role in reducing neuroinflammation by inhibiting the sumoylation of Tom1 and facilitating microglial migration. CIH reduces SENP1 levels, thereby inhibiting migration and contributing to neuroinflammation and neural injury (Figure 2) [27]. Microglia‐mediated neuroinflammation is a hallmark of numerous neurodegenerative diseases [52]. Understanding these mechanisms is essential for developing treatment for CIH‐induced neurodegenerative conditions involving activated microglia.

#### 2.2.3 Oligodendrocyte

Oligodendrocytes are a type of glial cell in the CNS primarily responsible for forming the myelin sheaths that insulate nerve fibers, facilitating proper neuronal signal transduction and brain function [53]. Myelin sheath is a multilayer lipid membrane structure, which can wrap around axons, ensuring the efficient transmission of action potentials. This process is crucial for accelerating impulse conduction and enhancing the overall efficiency of neural communication [54]. CIH has a range of impacts on oligodendrocytes and is potentially involved in the pathogenesis of several neurological diseases. For example, CIH may upregulate genes associated with oxidative phosphorylation and oxidative stress in oligodendrocytes by influencing microRNA expression, histone modification, and DNA methylation. These changes may result in oxidative damage to oligodendrocytes, contributing to neuroinflammation and neurodegenerative disease [55]. Additionally, CIH can stimulate the production of ROS in oligodendrocytes by activating microglia‐induced oxidative stress, while simultaneously reducing ATP production. This oxidative stress can lead to oligodendrocyte damage and demyelination, which plays an important role in the pathological process of MS (Figure 2) [28, 55]. A deeper understanding of how CIH impacts oligodendrocytes through these mechanisms will provide valuable insights into the underlying causes of related diseases and offer potential avenues for the development of new treatments.

#### 2.2.4 Advances in the Research of CIH in Neurological Diseases

CIH plays a role in the pathogenesis of various neurodegenerative diseases by affecting glial cells, particularly microglia and astrocytes. Studies have shown that CIH can participate in the progression of conditions such as AD, PD, MS, cerebral ischemia, and hypertension through its effects on glial cell function [54, 56, 57]. With further research, a deeper understanding of how CIH influences glial cells in the CNS may pave the way for the development of new therapeutic strategies to treat neurodegenerative diseases.

## 3 Therapeutic Strategies and Future Directions

### 3.1 Research Progress of CIH in Related Neurological Diseases

#### 3.1.1 Alzheimer's Disease

Alzheimer's disease (AD) is a leading cause of dementia and is rapidly becoming one of the most deadly and burdensome diseases in this century. It results from the progressive degeneration and death of neural cells, leading to memory loss, cognitive impairment, behavior, and psychological symptoms [58]. The characteristic pathological changes of AD include amyloid β plaque accumulation and neurofibrillary tangles formed by the abnormal phosphorylation of tau protein. Clinically, AD manifests as memory impairment, cognitive decline, language disorder, visuospatial skills damage, and emotional and behavioral changes, ultimately impairing daily living abilities [59]. Epidemiological studies show that more than 50% of AD patients suffer from sleep apnea [60]. Increasing evidence suggests a close relationship between CIH and AD. Chronic hypoxia contributes to neuronal and glial cell damage, which is central to AD pathogenesis [20, 61, 62]. CIH exacerbates AD by activating microglia to trigger neuroinflammation, inducing astrocytic proliferation, promoting pathological tau protein accumulation, impairing synaptic plasticity, and influencing AD‐related gene expression [14, 20, 39, 51, 63].

Currently, there is no definitive treatment to halt AD progression. Therapies focus on delaying disease progression, improving symptoms, and maintaining the quality of life through pharmacological interventions, cognitive training, behavior therapy, and supportive treatment [58]. For AD cases influenced by CIH, interventions such as CPAP, anti‐inflammatory drugs, antioxidants, and neuroprotective agents may mitigate CIH‐induced neuroinflammation and cognitive decline. Compounds like SMND‐309 ( *Salvia miltiorrhiza* extract), pterostilbene (Pte), melatonin, and huperzine A‐liposomes have shown promise in reducing CIH‐induced damage in AD patients. These agents exert their protective effects through distinct yet complementary mechanisms: SMND‐309 and Pte primarily attenuate CIH‐mediated damage via potent anti‐inflammatory and antioxidant activities; melatonin modulates amyloid pathology by suppressing β‐amyloid production and aggregation, while huperzine A‐liposomes provide dual protection through antioxidant effects and mitigation of iron‐induced oxidative stress [56, 57, 64, 65]. Memantine is a noncompetitive N‐methyl‐D‐aspartate (NMDA) receptor antagonist that treats moderate to severe Alzheimer's disease by regulating glutamatergic neurotransmission (Table 1) [66]. These insights into CIH's role in AD provide potential targets for developing new treatment strategies.

**TABLE 1:** Pharmacologic approaches for regulating CIH‐induced neurodegenerative diseases.

Neurodegenerative diseases	Targets	Drugs	References
Alzheimer's disease	Antioxidant	SMND‐309	Li et al. [56]
	Anti‐inflammatory	Pterostilbene	Liu et al. [57]
	Reduce hippocampal β‐amyloid generation	Melatonin	Ng et al. [65]
	Protect neuron	Huperzine A‐Liposomes	Yang et al. [64]
	NMDA receptor antagonists	Memantine	Jarvis et al. [66]
Parkinson's disease	Dopamine prodrug	Levodopa	Fox et al. [67]
	Dopamine agonists	Pramipexole	Mizuno et al. [68]
		Ropinirole	
	MAO‐B inhibitor	Rasagiline	Fox et al. [67]
		Selegiline	
	COMT inhibitor	Entacapone	Liao et al. [69]
	Anticholinergic drugs	Trihexyphenidyl	Brocks et al. [70]
		Benzatropine	
Cerebral apoplexy	Anti‐inflammatory	NSAIDs	Shehjar et al. [71]
		Glucocorticoid	
	Neuroprotectant	Diazepam	Liu et al. [72]
		Curcumin	Marques et al. [73]
		Resveratrol	Singh et al. [74]
	Antioxidant	Vitamin C	Tang et al. [75]
	Vasodilator	Amlodipine	Balligand et al. [76]
	Antiplatelet drugs	Aspirin	Chen et al. [77]
	Tissue plasminogen activator (tPA)	Alteplase	Pan et al. [78]
	Stem cell therapy	—	Xu et al. [79]
	Improve cerebral blood flow and angiogenesis	DM199	Alexander‐Curtis et al. [80]
Multiple sclerosis	β‐interferon drugs	Avonex	Cohan et al. [81]
		Betaferon	Etemadifar et al. [82]
	—	Glatiramer acetate	Boster et al. [83]
	—	Teriflunomide	Oh et al. [84]
	—	Fingolimod	Chun et al. [85]
	Target cell adhesion molecule A4 integrin	Natalizumab	Morrow et al. [86]
	—	Alemtuzumab	Riera et al. [87]

#### 3.1.2 Parkinson's Disease

Parkinson's disease (PD) is the second most prevalent neurodegenerative disorder after AD, affecting over 6 million people globally [88]. Pathologically, PD is marked by Lewy bodies and Lewy neurites, as well as the loss of dopaminergic neurons in the substantia nigra and other brain regions. Clinically, it manifests through motor and non‐motor symptoms (NMS). Motor symptoms include bradykinesia, static tremors, muscle stiffness, and postural or gait abnormalities. Non‐motor symptoms, which affect nearly all PD patients, include sensory deficits, constipation, urinary dysfunction, orthostatic hypotension, memory loss, depression, pain, and sleep disorders [89].

Epidemiological studies have shown that 20%–60% of PD patients also suffer from obstructive sleep apnea (OSA), and OSA‐induced CIH is related to the worsening cognitive and motor symptom dysfunction in PD patients [59, 90]. α‐synuclein, a presynaptic protein, plays a pivotal role in PD pathology by forming Lewy bodies [91]. CIH can induce the overexpression and oligomerization of α‐synuclein, while phosphorylated α‐synuclein contributes to PD progression by disrupting endocytic vesicles and undergoing ubiquitination [41]. Moreover, CIH induces oxidative stress and inflammation in brain regions like the substantia nigra and hippocampus, which are implicated in early PD pathology [19].

Though there is no cure for PD, various treatments can alleviate symptoms, including pharmacological therapies, surgical treatment, psychotherapy, and rehabilitation. Medications include dopamine precursors, dopamine agonists, MAO‐B inhibitors, COMT inhibitors, anticholinergics, and NMDA receptor antagonists [92]. Levodopa is a dopamine prodrug that alleviates symptoms by directly supplementing dopamine [67]. Pramipexole and ropinirole are dopamine agonists that improve motor symptoms in Parkinson's disease patients by activating dopamine receptors [68]. Rasagiline and selegiline are MAO‐B inhibitors that exert a comprehensive therapeutic effect by inhibiting MAO‐B enzymes, prolonging the action time of dopamine, reducing glutamate release, and blocking sodium channels [67]. Entacapone is a COMT inhibitor that improves motor symptoms and quality of life in Parkinson's disease patients by inhibiting COMT enzyme activity, prolonging the half‐life of levodopa, and enhancing its action time in the central nervous system [69]. Trihexyphenidyl and Benzatropine are anticholinergic drugs that block M‐type cholinergic receptors in the central nervous system, reduce acetylcholine neurotransmission, correct the imbalance between dopaminergic and cholinergic pathways, and effectively alleviate symptoms of Parkinson's disease (Table 1) [70]. While these treatments address PD symptoms, they do not specifically target CIH. Current research on CIH‐related drug interventions for PD is limited, and no significant therapeutic benefits have been observed yet. However, with continued exploration of CIH's mechanisms in PD, novel, targeted treatments are expected to emerge in the future.

#### 3.1.3 Cerebral Apoplexy

Cerebral apoplexy, also known as stroke or cerebrovascular accident, is an acute cerebrovascular event caused by the sudden rupture or blockage of brain blood vessels, resulting in restricted blood and subsequent brain tissue damage [93]. Strokes are classified into two major types: ischemic and hemorrhagic. Ischemic stroke occurs due to blood vascular obstruction, while hemorrhagic stroke results from a ruptured blood vessel [94, 95]. In adults, ischemic strokes are more common, accounting for 60%–70% of all cases, whereas in children, hemorrhagic and ischemic strokes occur at roughly equal rates [96]. Symptoms of stroke include sudden loss of balance, dizziness, slurred speech, drooling, facial droop, aphasia, severe headache, weakness, mobility issues, and, in severe cases, coma [94].

OSA is not only a significant factor for stroke, but also a frequent complication post‐stroke, often worsening outcomes [97]. Mechanisms like CIH, sleep fragmentation, and intrathoracic pressure fluctuation caused by OSAHS are considered to mediate cardiovascular risks. These effects hinder stroke recovery by reducing brain oxygenation, inducing white matter damage, and altering functional brain connectivity [98]. Research suggests that CIH activates microglia and elevates pro‐inflammatory cytokine production, contributing to neuronal damage and increasing stroke risk [99]. However, the precise mechanism remains unclear, warranting further investigation.

Stroke is a serious public health issue, with treatment typically including activity‐based therapies, pharmacological interventions, and cell‐based approaches [100]. Drug‐based treatments include tissue plasminogen activator (tPA), γ‐aminobutyric acid (GABA) receptor agonists, glutamate receptor antagonists, sodium and calcium channel blockers, fibrinogen consumption agents, and DM199 [71]. Treatment strategies for stroke related to CIH center around multiple key aspects. These include alleviating neuroinflammation, strengthening neuroprotection mechanisms, optimizing cerebral blood flow, and modulating the physiological and pathological processes associated with CIH. Anti‐inflammatory treatments include nonsteroidal anti‐inflammatory drugs (e.g., ibuprofen, aspirin) and glucocorticoids, while neuroprotective agents involve glutamate receptor antagonists and GABA receptor agonists [71, 73, 74, 101, 102, 103, 104]. Alteplase, a tissue plasminogen activator (tPA), exerts thrombolytic effects in stroke treatment through fibrin‐specific fibrinolysis, restoration of cerebral perfusion, attenuation of inflammatory responses, and blood–brain barrier preservation [78]. Diazepam, a GABA receptor agonist, confers neuroprotection against ischemic injury by suppressing neuronal hyperexcitation and attenuating ischemia‐induced intracellular calcium overload [72]. Curcumin and resveratrol exert neuroprotective effects in stroke through anti‐inflammatory, antioxidant, and neuroregenerative mechanisms [73, 74]. Amlodipine, a calcium channel blocker, prevents stroke through vasodilation‐mediated blood pressure reduction [76]. Aspirin, an antiplatelet agent, prevents stroke occurrence and recurrence through platelet aggregation inhibition and thrombus reduction (Table 1) [77]. As research deepens our understanding of CIH's role in stroke, more effective pharmacological treatments are on the horizon.

#### 3.1.4 Multiple Sclerosis

Multiple sclerosis (MS) is a chronic autoimmune disease characterized by inflammatory damage to the myelin sheath, the protective covering of the nerves in the brain and spinal cord. This damage disrupts the transmission of nerve signals, leading to various symptoms such as fatigue, blurred vision, and eye pain (optic neuritis), weakness or sensory changes in specific parts of the body (e.g., face, arms, or legs), dizziness, balance problems, memory or cognitive issues, and bladder control difficulties. MS patients are also more prone to depression and anxiety, further impacting their quality of life and increasing disability risk [105].

Although there is no definitive epidemiological data linking OSAHS to MS, studies have pointed out that OSAHS may exacerbate clinical manifestations in MS patients. The mechanism by which OSAHS influences MS may involve CIH, which increases oxidative stress and inflammation, disrupts neurotransmitter balance, and ultimately leads to neuronal damage [42].

In recent years, significant advancements have been made in the treatment of MS. Drug therapies include interferon β, glatiramer acetate (GA), teriflunomide, dimethyl fumarate, fingolimod, natalizumab, and alemtuzumab [106, 107]. Avonex and Betaferon, interferon β formulations, attenuate central nervous system damage through anti‐inflammatory and immunomodulatory mechanisms [81, 82]. Glatiramer acetate and teriflunomide, immunomodulatory agents, alleviate multiple sclerosis via anti‐inflammatory and neuroprotective actions [83, 84]. Fingolimod, a sphingosine‐1‐phosphate (S1P) receptor modulator, alleviates multiple sclerosis through dual immunomodulatory and neuroprotective effects mediated by S1P receptor regulation [85]. Natalizumab, a selective adhesion molecule inhibitor, mitigates multiple sclerosis progression by inhibiting α4 integrin‐mediated inflammatory cell migration into the central nervous system [86]. Alemtuzumab, a humanized monoclonal antibody, attenuates multiple sclerosis progression through CD52‐targeted depletion of T and B cells, thereby reducing central nervous system inflammation and demyelination (Table 1) [87]. While no specific drugs have been developed for CIH‐induced MS, treatments like CPAP, along with anti‐inflammatory and antioxidant medications, are used to manage CIH‐induced symptoms in MS patients.

#### 3.1.5 Huntington's Disease and Amyotrophic Lateral Sclerosis

Huntington's disease (HD) is an autosomal dominant neurodegenerative disorder characterized by progressive motor dysfunction, cognitive decline, and psychiatric disturbances. Its hallmark symptoms include chorea, dystonia, incoordination, cognitive deterioration, and behavioral difficulties. The pathogenesis of HD is linked to the extension of the CAG repeat sequence in the Huntington gene, resulting in polyglutamine chain elongation that disrupts normal protein function and induces neurotoxicity [108].

Amyotrophic lateral sclerosis (ALS) is a fatal neurodegenerative disorder that targets both the upper motor neurons (UMN) and lower motor neurons (LMN). This leads to progressive muscle weakness, which impairs voluntary movement, including limb function, swallowing (dysphagia), speech (dysarthria) and eventually respiratory failure. ALS primarily affects the brainstem, cervical, thoracic, and lumbar segments of the nervous system [109].

At present, there is no cure for either HD or ALS. This underscores the critical need for additional clinical investigation. However, CIH interventions, including pharmacological and non‐pharmacological approaches, offer novel strategies for modifying HD and ALS progression [110, 111]. Though there is no definitive evidence directly linking OSAHS with HD or ALS, recent studies suggest that CIH may exacerbate the pathological processes of HD and ALS by activating poly (ADP ribose) polymerase family member 1 (PARP1) [112]. In summary, HD and ALS represent progressive neurodegenerative disorders currently lacking curative treatments. While CIH may exacerbate disease pathology through PARP1 activation, the underlying mechanisms require further elucidation. Future investigations should prioritize delineating the role of PARP1 in HD and ALS pathogenesis to inform novel therapeutic approaches.

### 3.2 The Role of CIH in Neural Injury: Implications for Drug Development in Neurological Disorders and Future Therapeutic Prospects

#### 3.2.1 The Importance of CIH Research for Advancing Neurological Drug Development

CIH is a key pathological feature of OSA. This condition exposes patients to repeat cycles of hypoxia and reoxygenation during sleep, which can profoundly impact the nervous system. These fluctuations in oxygen levels may contribute to or exacerbate various neurological conditions, such as cognitive dysfunction and neurodegenerative diseases [2, 12]. While previous sections have outlined therapeutic interventions for various neurological disorders, the efficacy of existing pharmacological approaches may be compromised in the context of chronic intermittent hypoxia (CIH)‐induced neurological pathologies. This underscores the necessity for further investigation and therapeutic optimization specifically targeting CIH‐associated neurological conditions (Table 1). Research on CIH helps us gain insights into the pathogenesis of these conditions, especially in relation to oxidative stress, inflammation, neuronal apoptosis, and neurotransmitter imbalance [14, 21]. For example, SUMO‐specific protein 1 (SENP1) has been found to alleviate CIH‐induced neuronal injury by inhibiting the TOM1 pathway [27]. Tanshinone IIA promotes autophagy via the AMP‐activated protein kinase (AMPK)‐mechanistic target of the rapamycin (mTOR) signaling pathway, while Banxia Houpu decoction (BHD) reduces CIH‐induced neuronal damage by inhibiting ferroptosis [25, 113]. By investigating how CIH affects the nervous system, researchers can identify novel biomarkers and therapeutic targets, potentially leading to the development of more effective treatment strategies and medications.

#### 3.2.2 Emerging Therapeutic Targets and Prospects for CIH‐Related Neural Injury Research

As one of the main pathological features of OSAHS, CIH plays a very important role in the risk of neurodegenerative diseases in OSAHS patients. Current research on therapeutic strategies for CIH‐induced neural injury centers on several key areas.

First, neuroprotective strategies aim to minimize damage and promote nerve regeneration. This includes using factors such as osteopontin (Spp1), insulin‐like growth factor 1 (IGF‐1), and viral overexpression of cystic fibrosis factor (Cntf) (AAV‐OIC) to activate the intrinsic growth capacity of neurons [114, 115, 116]. Moreover, delayed delivery of fibroblast growth factor 2 (FGF‐2) and epidermal growth factor (EGF) helps create a supportive matrix for axon growth in damaged areas, while glial cell line‐derived neurotrophic factor (GDNF) attracts regenerating axons to their natural target [116].

Second, new therapeutic methods are being explored, such as neuromodulation techniques like electrical or chemical stimulation, noninvasive methods like transcranial magnetic stimulation (TMS) and transcranial direct current stimulation (tDCS), and photobiomodulation therapy (PBMT) [117, 118, 119, 120]. Electrical stimulation aids nerve cell recovery by continuously stimulating neurons, while hydrogen sulfide, known for its cytoprotective properties, can induce a low metabolic state to combat hypoxia or ischemia‐induced apoptosis. TMS and tDCS can alter neural activity by adjusting the excitability of neurons, helping them adapt to CIH‐related changes [121]. PBMT, also known as low‐level laser therapy (LLLT), employs specific light wavelengths to stimulate biological tissues. Its mechanism involves photon energy interacting with intracellular photoreceptors, enhancing cellular metabolism and repair [120]. This interaction can facilitate nerve regeneration, alleviate inflammation, and bolster the plasticity of neural networks, exhibiting vast potential applications in neural regulation [122, 123, 124].

Third, the field of biomaterials and tissue engineering is investigating the use of conductive hydrogels to facilitate nerve regeneration. These hydrogels, composed of conductive materials like metal nanoparticles and conductive polymers, mimic the electrical signal environment of the body, promoting neural repair and regeneration. Additionally, they can also release bioactive substances such as neurotrophic factors to enhance neural cell proliferation and differentiation [125].

Finally, the development of specific drugs that target CIH‐induced pathways, such as the microglia‐mediated inflammatory pathway, P_2_X_7_ receptor‐mediated neurotoxicity, and oxidative stress pathways, is a key focus [116]. Research into these pathways not only deepens our understanding of the mechanisms behind CIH‐induced neural injury but also serves as a vital reference for the development of new treatment strategies [28, 32, 35].

## 4 Conclusion and Outlook

The precise mechanisms underlying CIH‐induced neural injury remain unclear. One challenge is the variation in CIH models used by different researchers, with significant differences in hypoxia exposure time, duration, and oxygen concentration. These inconsistencies reduce the comparability and reproducibility of experimental results, making it difficult to generalize findings from animal and cellular studies to humans. Additionally, current CIH models primarily simulate chronic intermittent hypoxia but fail to account for other aspects of OSAHS, such as sleep fragmentation and microarousals, which limits their ability to fully replicate the neural injury mechanisms of OSAHS [126].

While some pathways involved in CIH‐induced neural injury have been identified, the exact mechanisms remain elusive, and further investigation is required to confirm and expand on these findings. In summary, to deepen our understanding of CIH‐induced nerve damage and develop effective treatment strategies, it is crucial to standardize CIH models, improve simulation conditions, and conduct in‐depth research on key targets in known pathways.

As our understanding of the pathophysiological mechanisms behind CIH and neural injury advances, the development of targeted therapies becomes increasingly viable. This progress offers strong hope that future treatments for OSAHS‐related neural injury will be more effective and personalized, leading to significant improvements in patients' quality of life and reducing the associated health risks. With continued research, we can anticipate more precise interventions tailored to individual patient needs, ultimately offering better management and outcomes for these comorbid conditions.

## Author Contributions

Writing – original draft: Nan‐Nan Jia. Writing – review and editing: Chen Qiao and Mei‐Juan He. Methodology: Nan‐Nan Jia, Meng‐Fan Yao, and Chun‐Xue Zhu. Investigation: Han‐Peng Huang, Hai‐Feng Zhu, and Zun‐Yu Chen. Project administration: Chen Qiao and Han‐Peng Huang. Resources: Chen Qiao and Han‐Peng Huang.

## Conflicts of Interest

The authors declare no conflicts of interest.

## Data Availability

The data that support the findings of this study are available from the corresponding author upon reasonable request.
